# The effectiveness of a web-based brief alcohol intervention in reducing heavy drinking among adolescents aged 15–20 years with a low educational background: a two-arm parallel group cluster randomized controlled trial

**DOI:** 10.1186/1471-2458-13-694

**Published:** 2013-07-30

**Authors:** Carmen V Voogt, Marloes Kleinjan, Evelien AP Poelen, Lex ACJ Lemmers, Rutger CME Engels

**Affiliations:** 1 Radboud University Nijmegen, Behavioural Science Institute, P.O. Box 9104, 6500, HE Nijmegen, the Netherlands; 2 Trimbos Institute, Netherlands Institute of Mental Health and Addiction, Utrecht, the Netherlands

**Keywords:** Web-based brief alcohol intervention, Heavy drinking, Adolescents, Low educational background

## Abstract

**Background:**

To evaluate the slightly modified version of the web-based brief alcohol intervention “What Do You Drink” (WDYD) among heavy drinking adolescents and young adults aged 15–20 years with a low educational background at one and six months follow-up.

**Methods:**

A two-arm parallel group cluster randomized controlled trial was conducted online in the Netherlands in 2011–2012. Participants included in the trial were recruited from preparatory and secondary vocational education institutions and had to be between 15 and 20 years of age and report heavy drinking in the past six months. In total, 73 classes representing 609 (59.9% male) participants were allocated to the experimental condition (37 classes, 318 participants: WDYD intervention) or control condition (36 classes, 291 participants: no intervention). Outcomes were heavy drinking, weekly alcohol consumption, and frequency of binge drinking.

**Results:**

Regressions analyses revealed no significant main intervention effects on any of the alcohol outcomes at one and six month’s follow-up according to the intention-to-treat principle. Additionally, there were no moderating effects of gender, age, educational level, and readiness to change on the relation between the WDYD intervention and the alcohol outcomes at follow-up.

**Conclusions:**

The WDYD intervention was not effective in reducing alcohol consumption among heavy drinking adolescents and young adults aged 15–20 years with a low educational background at one and six months follow-up. However, the absence of intervention effectiveness cannot be used as an argument for not conducting these types of interventions with low educated individuals, since our study was the first to target this population.

**Trial registration:**

Netherlands Trial Register NTR2971

## Background

Heavy drinking is a serious burden on health and economy in most Western countries and contributes to 4% of total mortality. Additionally, economic costs attributable to heavy drinking in Western countries are generally more than 1% of the gross domestic product [1]. The low-risk drinking guidelines of the Dutch National Health Council recommend that adults should not drink more than one (for females) or two (for males) glasses of standard alcohol units per day, with one unit representing ten grams of ethanol, and adolescents under the age of 18 should abstain from alcohol [2]. However, alcohol consumption estimates in the Netherlands indicate that a substantial proportion of adolescents and young adults drink above these guidelines and contribute to heavy drinking. This means drinking more than 7 (girls aged 15–16 years), 12 (boys aged 15–16 years), 14 (females aged 17–20 years), or 21 (males aged 17–20 years) glasses of standard alcohol units per week and/or drinking 5 or more glasses of standard alcohol units on one drinking occasion at least once per month for boys and girls aged 15–16 years and at least once per week for males and females aged 17–20 years. Heavy drinking is especially prevalent among 15 to 20 years old individuals with a low educational background [3-5]. The prevalence of heavy drinking among adolescents and young adults with a low educational background is alarming in light of the evidence showing that heavy drinking is associated with negative short and long-term health related consequences, academic performances, and social relationships [6,7]. Thus, it is necessary to develop interventions to decrease the number of adolescents and young adults and adolescents in general, and those with a low educational background in particular, who engage in heavy drinking, especially since they start drinking at a younger age, and engage in heavy drinking more often compared to higher educated peers [5,8,9]. Moreover, the prevalence rates of heavy drinking increase even more as adolescents get older [10]. Nonetheless, Dutch alcohol prevention and intervention programs targeting specifically adolescents and young adults with low education levels are practically non-existent [11,12].

Web-based brief alcohol interventions, consisting of a screening procedure and personalized feedback, are found to be effective in reducing heavy drinking in adolescents and young adults [13-16]. The general aim of these types of interventions is to reduce alcohol consumption patterns by providing discrepant personal information to increase individual’s motivation to change behaviour [17]. Personal drinking profile, risk factors, and normative comparisons are topics that are usually addressed in the screening procedure and when constructing the personalized feedback. The inclusion of normative comparisons of personal drinking levels and drinking levels of a relevant comparison group to correct misperceptions of descriptive drinking norms is based on social influence models [18]. Personalized normative feedback has been identified as an effective component of web-based brief alcohol interventions aimed at reducing heavy drinking [19,20], and it is commonly delivered in a non-judgmental, non-confrontational, and non-aversive manner to conform to Motivational Interviewing principles [21]. The high accessibility, convenience, and cost-effectiveness of web-based brief alcohol interventions [22] make these types of interventions suitable for targeting adolescents and young adults.

We are unaware of any studies examining the effectiveness of web-based brief alcohol interventions among adolescents and young adults with a low educational background. Most randomized controlled trials on web-based brief alcohol interventions targeted young adults attending higher education colleges or universities, possibly because these types of interventions require moderately high levels of reading and computer literacy [23]. In addition, evaluating intervention effectiveness among adolescents and young adults with a low educational background might be challenging, since they are difficult to recruit, and they tend to drop out of the intervention more frequently, resulting in low retention rates compared to higher educated groups [24]. Nonetheless, web-based brief alcohol interventions might also effectively reduce heavy drinking among adolescents and young adults with a low educational background for several reasons. First, a high prevalence of heavy drinking among adolescents and young adults with a low educational background constitutes a serious burden on health and economy. Thus, due to the absence of Dutch alcohol prevention and intervention programs targeting this population, there is a need to develop interventions. Furthermore, web-based brief alcohol interventions have a number of advantages over traditional face-to-face interventions regarding accessibility, anonymity, and cost-effectiveness [22]. Finally, most young adults have access to the Internet and are actively using it [25].

The present study evaluated the slightly modified version of the web-based brief alcohol intervention “What Do You Drink” (WDYD) among heavy drinking adolescents and young adults aged 15–20 years with a low educational background at one and six months follow-up. The WDYD intervention is initially developed by using the Intervention Mapping (IM) protocol [26] to detect and reduce heavy drinking among young 18 to 24 years old adults attending higher education colleges or universities [27]. The original WDYD intervention was slightly modified in terms of usability (i.e., use of language) to target adolescents and young adults between the ages of 15–20 years with a low educational background. Part one of the WDYD intervention contains a screening procedure and personalized feedback based on the screening outcomes, whereas part two focuses on goal-setting, action planning, and reinforcing drinking refusal self-efficacy through providing tips to maintain drinking goals in situations in which it is hard to resist alcohol. The core elements of the WDYD intervention are based on principles of Motivational Interviewing [21] and parts of the I-Change model [28], in which knowledge, social norms, and self-efficacy are included as the most changeable determinants of behavioral change. It took about 20 minutes to complete the single session WDYD intervention.

Recently, the original version of the WDYD intervention was evaluated at one and six months follow-up among heavy drinking students aged 18–24 years attending higher education colleges or universities. Although, no significant main effects were found for alcohol outcomes at both follow-ups, the WDYD intervention appeared to be effective in lowering alcohol consumption for several subgroups of college students (i.e., contemplators, carnival^a^ participants, and those scoring high on problem drinking) at one month follow-up [29]. Based on the results of this previous trial, we hypothesized to find no significant main effects for the WDYD intervention on alcohol consumption at one and six months follow-up. In addition, based on our earlier study among college students, we hypothesized that exposure to the WDYD intervention would prevent an increase in alcohol consumption at one month follow-up compared to receiving no intervention among the specific subgroup of participants who score high on problem drinking. Therefore, we further explored whether gender, age, educational level (i.e., risk factors), and readiness to change (i.e., a theoretical relevant factor that is targeted in the WDYD intervention to induce its effect) moderated the effect of the WDYD intervention on alcohol consumption at both follow-ups. Moderating effects were explored to establish whether subgroups at higher risk might be more likely to derive benefit from the WDYD intervention compared to subgroups at lower risk as well as to confirm the positive effects of the intervention across subgroups. Gaining insight into subgroups that derive most benefit from an intervention helps target specific subgroups for the WDYD intervention [30-33].

## Methods

### Study design

A two-arm parallel group cluster randomized controlled trial was used to evaluate the effectiveness of the WDYD intervention. In total, 609 participants were randomized in the experimental (*n* = 318: WDYD intervention) or control condition (*n* = 291: no intervention).

### Participants and procedure

Education in the Netherlands is oriented towards the needs and background of the students. After attending elementary education, students go to one of the three types of secondary education: preparatory secondary vocational education (VMBO), senior general education (HAVO) and pre-university education (VWO). A VMBO education trains students for secondary vocational education (MBO) or, in some cases, to move on to HAVO. A HAVO education is a preparation for a higher professional education (HBO) or university, but students can also go to VWO. A VWO education prepares students for a university education. The present study recruited participants from VMBO and MBO institutions in 2011–2012.

The VMBO and MBO institutions were selected from a list of all educational institutions in different regions (i.e., Overijssel, Gelderland, Noord-Brabant, and Limburg) in the Netherlands by means of a convenience sampling strategy. The selected educational institutions received letters inviting them to participate in the study and containing information about the study and inclusion criteria. A cover story was used in which the institutions were informed that their students participated in a study examining newly developed health education materials addressing alcohol use. After two weeks, the VMBO and MBO institutions were contacted by telephone to establish whether they were willing to participate in the study. If they were willing to be involved in the study, they were requested to participate with as many classes as possible to recruit the necessary amount of participants based on the power calculation [34]. In addition, they were requested to distribute study invitation letters to the parents of students aged 15–16 years, giving them the opportunity to respond if they had any objections to their child’s participation. All students of the participating classes were followed during the entire study period to avoid stigmatization and social exclusion. Yet, after the recruitment and enrolment of the classes in the trial, all students filled in a baseline survey to establish whether they met the inclusion criteria of the study and could be included in the analyses. Inclusion criteria of the study were that participants needed to 1) be between 15 and 20 years of age, 2) report heavy drinking in the past six months, and 3) be ready to change their alcohol consumption. Participants who showed symptoms of alcohol abuse or dependence (i.e., an AUDIT score of 20 above [35]) and/or received treatment for alcohol-related problems were excluded from the study because the WDYD intervention focuses on the prevention of heavy drinking rather than problem drinking.

The power calculation was based on study findings of the effectiveness of a web-based personalized feedback intervention on heavy alcohol use in male adults in the Netherlands [36]. As reported in our study protocol, 750 participants were needed to detect an increase in the percentage of participants showing low-risk drinking of 42% (315/750) in the experimental condition versus 31% (232.5/750) in the control condition corresponding to a number needed to treat of 9 (i.e., moderate to large effect) at one month follow-up with a 2-sided test at alpha = 0.05, a power of (1-beta) = 0.80 and an anticipated dropout rate of 15% after randomization [34]. The power calculation accounted for the clustered nature of the data (participants are nested in classes) with expected intra-class correlation coefficients (ICC’s) between 0.03 and 0.06. In total, 1374 participants from 92 classes at 9 VMBO and 10 MBO institutions were recruited. This number was deemed sufficient to identify 750 heavy drinking students, since a previous report on alcohol consumption among students from 20 different MBO institutions in the Netherlands (*N* = 7.977) indicated that a total of 79.1% of participants drank at least one glass of alcohol per week, the average consumption was 5.1 glasses of alcohol per week and 63% of the population did not adhere to low-risk drinking norms^b^[37]. However, contrary to what was expected based on these prevalence rates among lower education students, 44% (*N* = 609) of participants in our sample could be classified as heavy drinkers. Of these 609 participants, only 63 indicated that they were ready to change their drinking behaviour. Due to the lack of readiness to change of the target population and time and financial constraints, we decided to include all heavy drinking adolescents and young adults aged 15–20 years (i.e., those meeting two out of the three inclusion criteria) in the study and run the analyses on 609 participants. To avoid contamination between the conditions, randomization using a computerized random number generator with blocked randomization scheme (block size 4) occurred by class level within the educational institutions. An independent researcher from the Behavioural Science Institute performed the allocation before baseline assessment. Participants were blinded to the aim of the study until the end of the study.

Surveys were administered online during school hours by means of school visits at baseline and at one and six months follow-up. The participants were assured anonymity and confidentiality, since the researchers were the only ones who had access to the data. The DVD workshop “Advertisement agency”, designed by the Trimbos Institute (Netherlands Institute of Mental Health and Addiction), was given as incentive by sending debriefing letters to the participating VMBO and MBO institutions at the end of the study period after the last measurements had been filled out. This workshop was developed for adolescents with an aim to focus on alcohol, tobacco, and drugs use as well as on peer pressure. No individual incentives were given. The Ethical Committee of the Faculty of Social Sciences at Radboud University Nijmegen approved the study [34]. This trial is registered in the Netherlands Trial Register (no. NTR2971).

### Interventions

Participants assigned to the experimental condition received the WDYD intervention. The content of the WDYD intervention is described in detail elsewhere [26,29]. Participants assigned to the control condition received no intervention.

### Outcomes^c^

#### Heavy drinking

Heavy drinking was defined as the percentage of participants drinking above the limits of low-risk drinking and assessed at baseline and one and six months follow-up [2]. This means drinking more than 7 (girls aged 15–16 years), 12 (boys aged 15–16 years), 14 (females aged 17–20 years), or 21 (males aged 17–20 years) glasses of standard alcohol units per week and/or drinking 5 or more glasses of standard alcohol units on one drinking occasion at least once per month for boys and girls aged 15–16 years and at least once per week for males and females aged 17–20 years [34].

#### Weekly alcohol consumption

The Dutch version of the Alcohol Weekly Recall [38] was used to assess weekly alcohol consumption at baseline and one and six months follow-up. Participants were asked to indicate retrospectively the exact number, size, and type of alcohol beverage they consumed on each day in the past seven days. Standardized responses were assured by providing an overview of standard units for various beverages. In total, 2.15% of the participants scored three standard deviations above the sample mean of weekly alcohol consumption and were given that value in order to retain outliers in the analyses (resulting range 0 to 62) [39].^d^

#### Frequency of binge drinking

Frequency of binge drinking was defined as the percentage of participants drinking 5 or more glasses of standard alcohol units on one drinking occasion at least once per month (boys and girls aged 15–16 years) or week (males and females aged 17–20 years) and assessed at baseline and after one and six months follow-up. Participants were asked how often they had drunk 5 or more glasses of standard alcohol units on one drinking occasion in the previous month or week, respectively. Responses were measured on an 8-point scale ranging from (0) “never” to (7) “every day” [29]. The definition of frequency of binge drinking was derived in different ways according to participants’ age since we assumed that the prevalence and effects would be too small in the youngest age group when using the "once per week" criterion for the total group. Therefore, we used the "once per month" criterion for 15–16 year olds and the "once per week" criterion for 17–20 year olds which is in line with a comparative study on the effectiveness of a web-based brief alcohol intervention among binge drinkers aged 15–20 years in the Netherlands [17].

#### Moderators

Gender (male vs. female), age (15–16 years vs. 17–20 years), educational level (VMBO vs. MBO), and readiness to change (no vs. yes) were explored as moderators and assessed at baseline. Participants’ readiness to change was assessed using one item asking participants, which of the following statement applied best to them: (1) “I do not drink alcohol anymore (action)”, (2) “In the future, I will keep drinking alcohol as much as I do now” (immotive), (3) “I want to reduce drinking alcohol in the future, but not within the upcoming six months (precontemplation)”, (4) “I want to reduce drinking alcohol within the upcoming six months” (contemplation), (5) “I want to reduce drinking alcohol within the upcoming month” (preparation), (6) “I have already reduced drinking alcohol, but less than six months ago” (action), and (7) “I have reduced drinking alcohol more than six months ago (maintenance)”. The question was developed to provide a short and easy to administer and score measure of participant's readiness to change that was also incorporated in the screening test of the WDYD intervention. Participants who selected statements 2 or 3 were considered not to be ready to change behavior (*n* = 508), whereas those who selected statements 1, 4, 5 or 6 were considered to be ready to change behavior or already in the process of changing behavior (*n* = 63) [40-42]. Readiness to change was dichotomized to be consistent with the moderation analyses in our previous study on the effectiveness of the WDYD intervention among heavy drinking college students aged 18–24 years up [29].

### Statistical methods

The intent-to-treat (ITT) principle and the completers-only framework were used to analyze all data. Missing data were handled by means of multiple imputations using the predictive mean matching method (MMS). In total, twenty imputed datasets were evaluated with *p* = 0.05 as a criterion for statistical significance by averaging the results (i.e., pooling). A completers-only framework was utilized with participants who participated in all measurements, without the inclusion of imputed data. Descriptive statistics were used to describe the baseline characteristics of the participants. Logistic regressions were conducted for heavy drinking and frequency of binge drinking, whereas linear regressions were conducted for weekly alcohol consumption to evaluate the effectiveness of the WDYD intervention at one and six months follow-up while adjusting for covariates that were unequally distributed across conditions at baseline. We reported 1) odds ratios (OR) and 95% confidence intervals (CI) for the dichotomous variables and 2) standardized coefficients (Betas), standard errors (SE), and p-values for the continuous variable. In addition, the effect sizes were calculated using Cohen’s d (i.e., M_1_ – M_2_ / √(SD_1_^2^ + SD _2_^2^) / 2) [43] for weekly alcohol consumption and numbers needed to treat (NNT) [44] for heavy drinking and frequency of binge drinking. ICC’s were calculated for all three dependent variables at one and six months follow-up to control for the clustered data since participants were nested within classes. Although ICC’s were expected to be between 0.03 and 0.06 [34], heavy drinking, frequency of binge drinking, and mean weekly alcohol consumption had mean ICC’s of 0.05, 0.04, and 0.12 at one month follow-up and 0.05, 0.05, and 0.07 at six months follow-up, respectively, indicating that class effect could be explain a part of the variance. Therefore, all regression analyses were adjusted for clustering and covariates in Mplus 6.0 [45]. In addition, all analyses were conducted using the maximum likelihood estimation with robust standard errors (MLR) to correct for the skewed distribution of the alcohol outcomes. Moreover, interaction terms were computed and entered into the regression models to examine differences in intervention effectiveness between subgroups at both follow-ups. Interaction terms were calculated as the products of the dummy coded intervention-control contrasts with gender, age, educational level, and readiness to change as moderators.

## Results

### Participant flow

**Figure 1:**
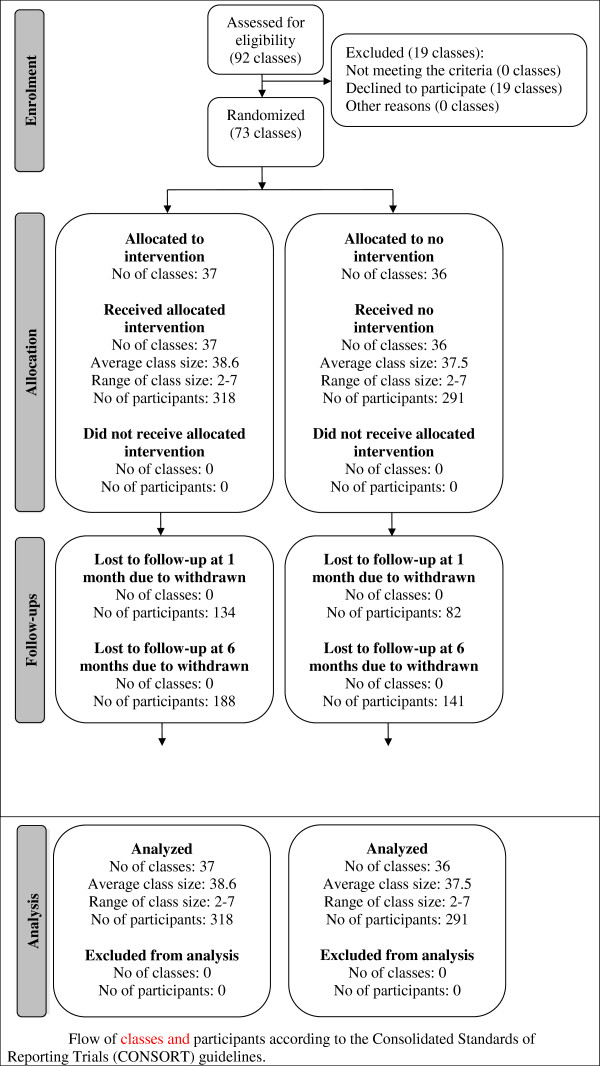
Flow of classes and participants according to the Consolidated Standards of Reporting Trials (CONSORT) guidelines.

Figure 1 illustrates the flow of the classes and participants. Overall, 92 classes representing 1374 participants were recruited and filled in the baseline survey of which 37 classes (*n* = 318) were allocated to the WDYD intervention and 36 classes (*n* = 291) allocated to no intervention. Due to sickness, truancy, or changing from educational institution, loss to follow-up rates were 35.5% at one-month follow-up and 54.0% at six months follow-up. Finally, 609 participants were heavy drinkers in the ages between 15–20 years and eligible for the intention-to-treat analyses, whereas 280 participants were eligible for the completers-only analyses.

### Baseline characteristics

The baseline characteristics at individual and class level for the experimental and control conditions are shown in Table 1. The sample had a mean age of 17.3 (*SD* = 1.3) and consisted of 59.9% males. Of the participants, 16.6% attended VMBO education, 36.5% drank heavily, and 63.4% engaged in binge drinking. The mean weekly alcohol consumption was 11.6 (*SD* = 13.7) glasses of standard alcohol units.

**Table 1:** Baseline characteristics at individual and cluster level

		**Intervention**	**Control**	**Total sample**
		**(*n*** **= 318)**	**(*n*** **= 291)**	**(*N*** **= 609)**
**Individual characteristics**				
Male, *n* (%)		217 (68.2)	148 (50.9)	365 (59.9)
Age, *M* (*SD*)		17.2 (1.3)	17.4 (1.2)	17.3 (1.3)
Education, *n* (%)				
	VMBO^a^	65 (20.4)	36 (12.4)	101 (16.6)
	MBO^b^	253 (79.6)	255 (87.6)	508 (83.4)
Readiness to change^c^, *n* (%)		36 (11.3)	27 (9.3)	63 (10.3)
Outcomes				
	Heavy drinking^d^, *n* (%)	117 (36.8)	105 (36.1)	222 (36.5)
	Frequency of binge drinking, *n* (%)	205 (64.5)	181 (62.2)	386 (63.4)
	Weekly alcohol consumption, *M* (*SD*)	12.0 (13.9)	11.3 (13.5)	11.6 (13.7)
Class characteristics				
Number		37	36	73
Size, *M* (*SD*)		38.6 (19.3)	37.5 (18.2)	38.1 (18.8)

*Note.N* number of participants, *M* mean, *SD* standard deviation. ^a^VMBO: preparatory secondary vocational education. ^b^MBO: secondary vocational education. ^c^Readiness to change alcohol consumption was assessed through one item asking the participants which statement applied best to them. Participants were considered to be ready to change alcohol consumption when they selected “I do not drink alcohol anymore” or “I want to reduce drinking alcohol within the upcoming six months” or “I want to reduce drinking alcohol within the upcoming month” or “I have already reduced drinking alcohol, but less than six months ago”. ^d^Drinking > 7 or 12 (girls/boys aged 15–16 years) or 14 or 21 (females/males aged 17–20 years) glasses of standard units of alcohol per week and/or drinking 5 or more glasses of standard alcohol units per occasion at least once per month for boys and girls aged 15–16 years and at least once per week for males and females aged 17–20 years (= binge drinking).

### Loss to follow-up

Attrition rates were 35.5% (*n* = 216, 134 in the intervention condition and 82 in the control condition) and 54.0% (*n* = 329, 188 in the intervention condition and 141 in the control condition) at one and six months follow-up, respectively, and related to conditions (*χ*^*2*^ = 12.9 [*df* = 1], *p* < .001 and *χ*^*2*^ = 7.0 [*df* = 1], *p* < .01). Attrition analyses on baseline variables and alcohol outcomes indicated that completers were more likely to be female (*χ*^*2*^ = 14.3 [*df* = 1], *p* < .001) and more likely to be younger (*t*(607) = 1.97, *p =* 0.05).

### Effect of the intervention

#### Heavy drinking and frequency of binge drinking

The results revealed no significant differences between conditions in heavy drinking and frequency of binge drinking at one and six months follow-up. The results were replicated in the completers-only analyses, with the exception of frequency of binge drinking at one month follow-up, indicating that 57.7% of the participants in the experimental condition drunk five or more glasses compared to 66.7% of the participants in the control condition (*OR* = 0.85; *CI* = 0.73 to 0.98; NNT = 11; *p* = 0.03) (see Table 2).

**Table 2:** Percentage of heavy drinking and frequency of binge drinking at one and six months follow-up by condition (WDYD intervention versus control): intention-to-treat analyses (multiple imputation) (N = 609) and completers-only analyses (n = 280) adjusted for clustering and covariates (i.e., gender and education level)

		**Intervention**		**Control**					
		***n***	***%***	***n***	***%***	***OR***	***95% CI***	***P***	***NNT***
***Heavy drinking***									
**1-month follow-up**									
	Intention-to-treat	318	25.3	291	26.3	0.96	[0.84 to 1.10]	0.54	83
	Completers-only	130	34.6	150	37.3	0.91	[0.79 to 1.04]	0.18	37
**6-month follow-up**									
	Intention-to-treat	318	29.5	291	31.5	0.97	[0.84 to 1.11]	0.65	49
	Completers-only	130	30.8	150	34.7	0.91	[0.77 to 1.08]	0.28	26
***Frequency of binge drinking***									
**1-month follow-up**									
	Intention-to-treat	318	43.3	291	47.7	0.92	[0.82 to 1.04]	0.18	23
	Completers-only	130	57.7	150	66.7	0.85	[0.73 to 0.98]	0.03	11
**6-month follow-up**									
	Intention-to-treat	318	55.3	291	57.5	0.95	[0.85 to 1.06]	0.35	49
	Completers-only	130	50.8	150	55.3	0.91	[0.80 to 1.03]	0.13	24

*Note.N* number of participants *OR* odds ratios, *CI* confidence interval, *P* p-value, *NNT* number needed to treat.

#### Weekly alcohol consumption

The findings showed no significant differences between the experimental and control condition in weekly alcohol consumption at both follow-up assessments. These findings were replicated in the completers-only analyses (see Table 3).

**Table 3:** Weekly alcohol consumption [standard deviations (SD)] at one and six months follow-up by condition (WDYD intervention versus control): intention-to-treat analyses (multiple imputation) (N = 609) and completers-only analyses (n = 280) adjusted for clustering and covariates (i.e., gender and education level)

		**Intervention**		**Control**						
		***M***	***SD***	***M***	***SD***	***dif***	***Beta***	***SE***	***P***	***Cohen’s d***
**1-month follow-up**										
	Intention-to-treat	13.2	16.1	12.3	15.0	0.9	−0.01	0.05	0.86	0.06
	Completers-only	13.0	16.2	10.8	12.2	2.2	−0.01	0.06	0.93	−0.02
**6-month follow-up**										
	Intention-to-treat	12.2	15.1	11.7	14.0	0.5	−0.01	0.05	0.89	0.00
	Completers-only	11.5	14.4	10.8	12.3	0.7	−0.01	0.06	0.83	0.08

*Note.N* number of participants, *M* mean, *SD* standard deviation, *Dif* difference in means between conditions, *SE* standard error. *P* p-value.

### Moderating effects

Moderation analyses revealed no significant moderating effects of gender, age, educational level, and readiness to change on the relation between the WDYD intervention and the alcohol outcomes, i.e., heavy drinking, frequency of binge drinking, and weekly alcohol consumption, at follow-up at one and six months after the intervention (results in Tables can be obtained from the first author upon request).

## Discussion

The present study evaluated the effectiveness of the slightly modified version of the web-based brief alcohol intervention “What Do You Drink” for adolescents and young adults aged 15–20 years with a low educational background using a two-arm parallel group cluster randomized controlled trial. As hypothesized, no significant main effects were found for the WDYD intervention on any of the alcohol outcomes at one and six months follow-up. In addition, the findings showed no moderating effects of gender, age, educational level, and readiness to change on the relation between the WDYD intervention and the alcohol outcomes at both follow-ups. In addition to the absence of main effects, also no moderating effects were found. An explanation for these latter null results could be found in the difficulties encountered in the recruitment process, which accounted for a smaller sample size (*N* = 609) than envisioned beforehand according to the power calculation (*N* = 750) [34]. Another factor that could explain the absence of moderating effects may be linked to the fact that low educated persons tent to respond better to visuals rather than text [46] with respect to online information and face more difficulties with interpreting and processing information [47]. It is debatable whether they have read, understood, and remembered the personalized feedback and normative comparisons with alarming content and utilized the tips to resist alcohol in high-risk drinking situations. The WDYD intervention may not be comprehensible and appealing enough to subgroups of heavy drinking adolescents and young adults attending VMBO and MBO education since they may not be stimulated enough to effectively process online information and increase their readiness to change alcohol consumption. Still, we modified the WDYD intervention in terms of usability to the target population, which indicated that its contents and design were appropriate. Moreover, it is reasonable to assume that the WDYD intervention (i.e., 20-minutes and one single-session) may not have been intensive enough to reduce alcohol consumption at follow-ups. Booster sessions might have increased students’ exposure to the WDYD intervention and thereby strengthen and/or extend intervention effects [48,49].

The abovementioned results should be considered in light of several limitations. First, we could not recruit the required number of participants as indicated by the power calculation. Additionally, the ICC’s were higher than expected in our study protocol [34], indicating that class effect could explain a part of the variance. Yet, in our regression models we adjusted for the nested data structure. Second, the attrition was relatively high in the present study, which is a common feature of many web-based delivered interventions also termed as “the law of attrition” [50]. Despite the attrition, the results established that, any difference between the experimental and the control condition on the alcohol outcomes at follow-ups was expected to be small and probably would not have reached statistical significance even with a larger sample size. In addition, the study had a low retention rate that was distributed unequally over both arms of the trial, indicating selective dropout. Yet, the results pertain to the intention-to-treat population. Acceptable participation and retention rates may be accomplished by providing significant (monetary) incentives employed in most trials [23] and/or by giving participants the opportunity to complete the surveys after school hours in the privacy of their homes. Third, a convenience sampling strategy was used; thus, participants did not have an equal chance of being selected; instead, they were selected based on availability, which may limit the generalisability. Fourth, participants in the control condition might have been exposed to the WDYD intervention if they had friends in the experimental condition who could have shared the information about the intervention. Yet, the contamination between conditions is expected to be small because the WDYD intervention is not yet available online and the randomization occurred at class level within the education institutions. The fifth limitation is the self-reported nature of the data, possibly resulting in social desirability or memory deficits that may have influenced the recall of alcohol consumption, which tends to decrease after two or three days [51-54]. Yet, possible underreporting of alcohol intake would be assumed to be equally present across both conditions. Finally, the present study did not consider the fluctuating nature of alcohol consumption among individuals [55-57] since it used only two follow-ups (i.e., one and six months), thereby increasing the danger of making inaccurate conclusions about intervention effectiveness. To obtain a higher precision in measuring intervention effectiveness and minimize the danger of inaccurate conclusions about intervention effectiveness when using few data time-points, employing ecological momentary assessments (EMA) might be an opportunity. This is a repeated sampling strategy to assess alcohol consumption in real-life settings at strategically selected moments in time [58]. Advantages of EMA are that it 1) can overcome shortcomings related to traditional methods of assessing alcohol consumption and intervention effectiveness, 2) uses refined outcome measures that are sensitive to change, which might alleviate sample size requirements, 3) enables one to examine whether intervention effectiveness on the treatment outcome is robust or varies over time when exploring multiple follow-ups while considering the fluctuating nature of alcohol consumption, and 4) can generate overall intervention effects that can help determine the time at which the intervention effects have stopped as well as the time at which “booster sessions” are needed [29]. The advantages of EMA justify the importance of adopting this method more widely in future randomized controlled trials on web-based brief alcohol interventions to measure intervention effectiveness.

## Conclusions

The WDYD intervention was not effective in reducing alcohol consumption at one and six months follow-up among heavy drinking adolescents and young adults aged 15–20 years with a low educational background. The absence of intervention effectiveness cannot be used as an argument for not conducting web-based brief alcohol interventions with adolescents and young adults with a low educational background, since our study was the first to target this population. Because of the limitations of the present study, more trials should be conducted, preferably by means of EMA, to test the effectiveness of web-based brief alcohol interventions for low educated individuals. In addition, future research should gain inside on methods that would be most effective in recruiting low educated individuals in trials or on characteristics of these individuals that are associated with successful participation and retention rates with an aim to improve the quality of research and help us better understand to obtain results.

### Endnotes

^a^Carnival is a four-day event celebrated in February before spring in the southern provinces in the Netherlands and characterized by excessive drinking patterns.

^b^Prevalence rates of heavy drinking according to our definition are not available for students attending VMBO and MBO education in the Netherlands.

^c^All outcomes pertained to the individual level.

^d^The extreme values were managed in the same way as we did in our previous study on the effectiveness of the WDYD intervention among heavy drinking college students aged 18–24 years [29].

## Competing interests

The authors declare that they have no competing interests.

## Authors’ contributions

CV is responsible for the data collection and data analysis, as well as for reporting the study results. All others authors are supervisors and grant applicators. All authors read and approved the final manuscript.

## Pre-publication history

The pre-publication history for this paper can be accessed here:

http://www.biomedcentral.com/1471-2458/13/694/prepub
